# The differentiation of Lgr5+ progenitor cells on nanostructures of self-assembled silica beads

**DOI:** 10.1371/journal.pone.0304809

**Published:** 2024-07-12

**Authors:** Wenjun Cai, Zhichun Huang, Baobin Sun, Ling Lu, Xiaoqiong Ding, Feng Tao

**Affiliations:** Department of Otorhinolaryngology-Head and Neck Surgery, Zhong Da Hospital, Southeast University, Nanjing, China; University of Colorado Boulder, UNITED STATES OF AMERICA

## Abstract

Supporting cells(SCs) have been demonstrated to be a reliable source for regenerating hair cells(HCs). Previous research has reported that Lgr5+ SCs can regenerate HCs both in vitro and in vivo. However, there is limited knowledge about the impact of the material on Lgr5+ cells. In this study, Lgr5+ cells were isolated from neonatal Lgr5-EGFP-CreERT2 transgenic mice by flow cytometry and then plated on self-assembled silica beads (SB). Lgr5+ cell differentiation was observed by immunofluorescence. We found that in the direct differentiation assay, the SB group generated more hair cells than the control group(*p < 0.05). Especially in the SB group, Lgr5+ progenitors generated significantly more Myo7a+ HCs outside of the colony than in the control group(**p < 0.01). In the sphere differentiation assay, we found that the diameter of spheres in the SB group was significantly larger compared to those of the control group(**p < 0.01). However, the difference in the ratio of myo7a+ cell counts was not obvious(P>0.05). The experiment proved that the self-assembled silica beads could promote the differentiation of Lgr5+ progenitors in vitro. Our findings implicate that nanostructures of self-assembled silica beads can be used as vectors for stem cell research in the inner ear.

## Introduction

According to the 2021 World Hearing Report, one-fifth of the global population is suffering from hearing loss. Sensorineural hearing loss is the most common type of hearing impairment [1]. Damage and loss of hair cells can lead to sensorineural hearing loss, so hair cell repair and regeneration is the key to hearing rehabilitation. Supporting cells(SCs) have been demonstrated to be a reliable source for regenerating hair cells. In mammals, the cochlear SCs in newborns contain HC progenitors and have a limited ability to regenerate HCs through direct differentiation and mitosis after injury [2–6]. Lgr5 is an epithelial cell protein first identified as a marker for intestinal stem cells and then shown to be essential for their function and recently proved to be expressed in cochlear supporting cells that surround the hair cells [4,7–9].

Leucine-rich repeat-containing G-protein coupled receptor 5 (Lgr5), a Wnt target gene, is a stable stem cell marker expressed in a subpopulation of cochlear SCs [10]. Wnt signaling is required for hair cell differentiation [11], which is increased by concurrent inhibition of Notch [2,11,12]. Recently research has shown that in neonatal mice, Lgr5+ cells are an HC progenitor population that can regenerate HCs [13]. The current study focuses on determining the molecular mechanisms underlying the HC regenerative capacity of Lgr5+ progenitor cells in the cochlea of newborn mice [14]. But the reaction of Lgr5+ progenitor cells toward the material has not been studied yet.

Research has shown that graphene can modulate the behavior of stem cells, including proliferation and differentiation [15–17]. Now we use the Self-Assembled Silica Beads with diameters of 500 nm and explore the effect of this material on inner stem cells. The silica beads were denoted as SB-500. Silica beads belong to the ultra-fine nanometer scale, with a size range from 1-100nm. They are non-toxic, tasteless, and pollution-free. The microstructure is spherical, flocculent, and reticular quasi-particle structure. Studies have shown that the self-assembled silica beads promote the growth of neurons, especially the silica beads with diameters ranging from 480 to 670 nm [18]. There have been many reports about neuron culture on the silica beads in vitro [19], but there have been no reports on the developmental responses of Lgr5+ cells.

In this study, Lgr5+ cells were separated by flow cytometry and then cultured on the self-assembled silica beads. This project focused on investigating the differentiation of Lgr5+ progenitor cells on Nanostructures of Self-Assembled Silica Beads.

## Materials and methods

### Mice and genotyping

We use neonatal Lgr5-EGFP-CreERT2 transgenic mice. It was used to report Lgr5 expression with EGFP tagging [20]. Genotyping of transgenic mice using genomic DNA from the toe was performed by adding 180 μl 50 mM NaOH, incubating at 98°C for 1 hour, and adding 20 μl 1M Tris-HCl. The primers for genotyping are as follows: Lgr5: (F) CTG CTC TCT GCT CCC AGT CT; wild-type (R) ATA CCC CAT CCC TTT TGA GC; mutant (R) GAA CTT CAG GGT CAG CTT GC; The protocol of genotyping as follows: 94°C for 5 minutes,94°C for 30 seconds,58°C 45 seconds,72°C for 45 seconds, go to step 2 for 32X, then 72°C for 10 minutes and 4°C for the end. All animal procedures were approved by the Animal Care and Use Committee of Southeast University.

### Material phenotype

**Fig 1:**
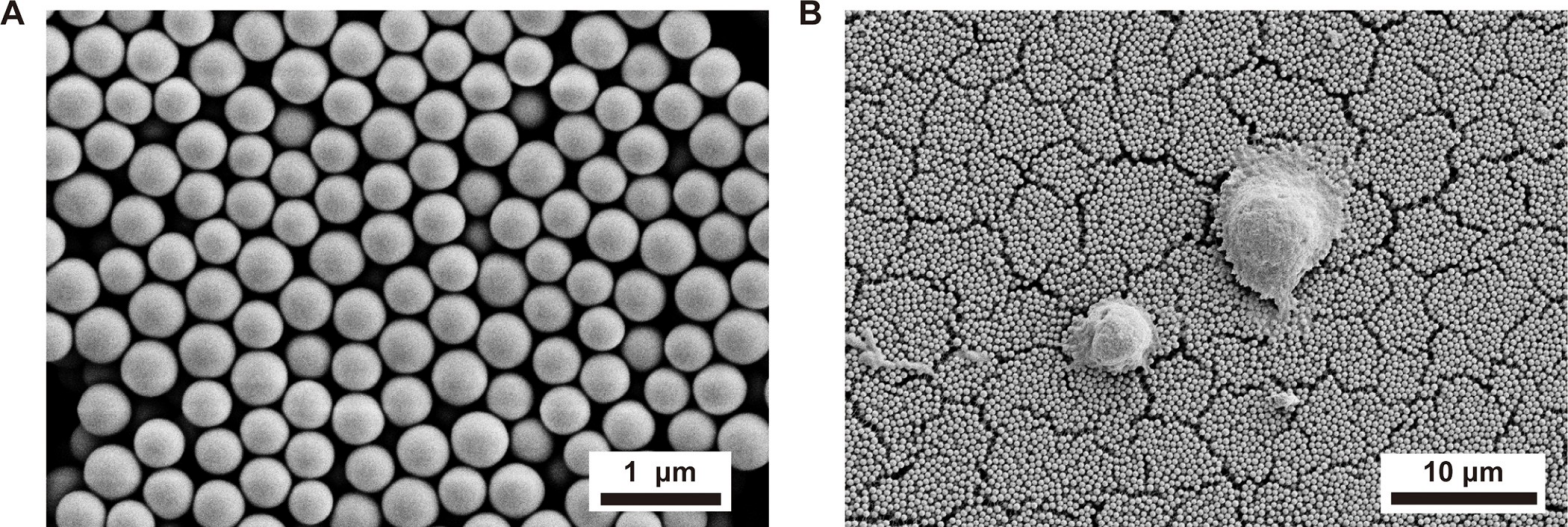
SEM images. (A)SEM images of the nanostructures assembled with SB-500. (B) SEM image of Lgr5+ progenitors cultured on SB-500.

The silica beads-500 were cleaned and centrifuged with 75% alcohol and sterile water. Then we spread two layers of SB-500 on slides. Finally, the slides were baked at 350°C for 4 hours. The Lgr5+cells cultured on the self-assembled silica beads were fixed in 2.5% glutaraldehyde solution overnight at 4°C. Then washed three times with 0.1 M PBS and a gradient of dehydration for alcohol. Promptly place the sample(including SB and cells plated on SB) in the desiccant for one day and then spray the sample with gold. Finally, the samples were observed by scanning electron microscope. (Fig 1)

### Flow cytometry

**Fig 2:**
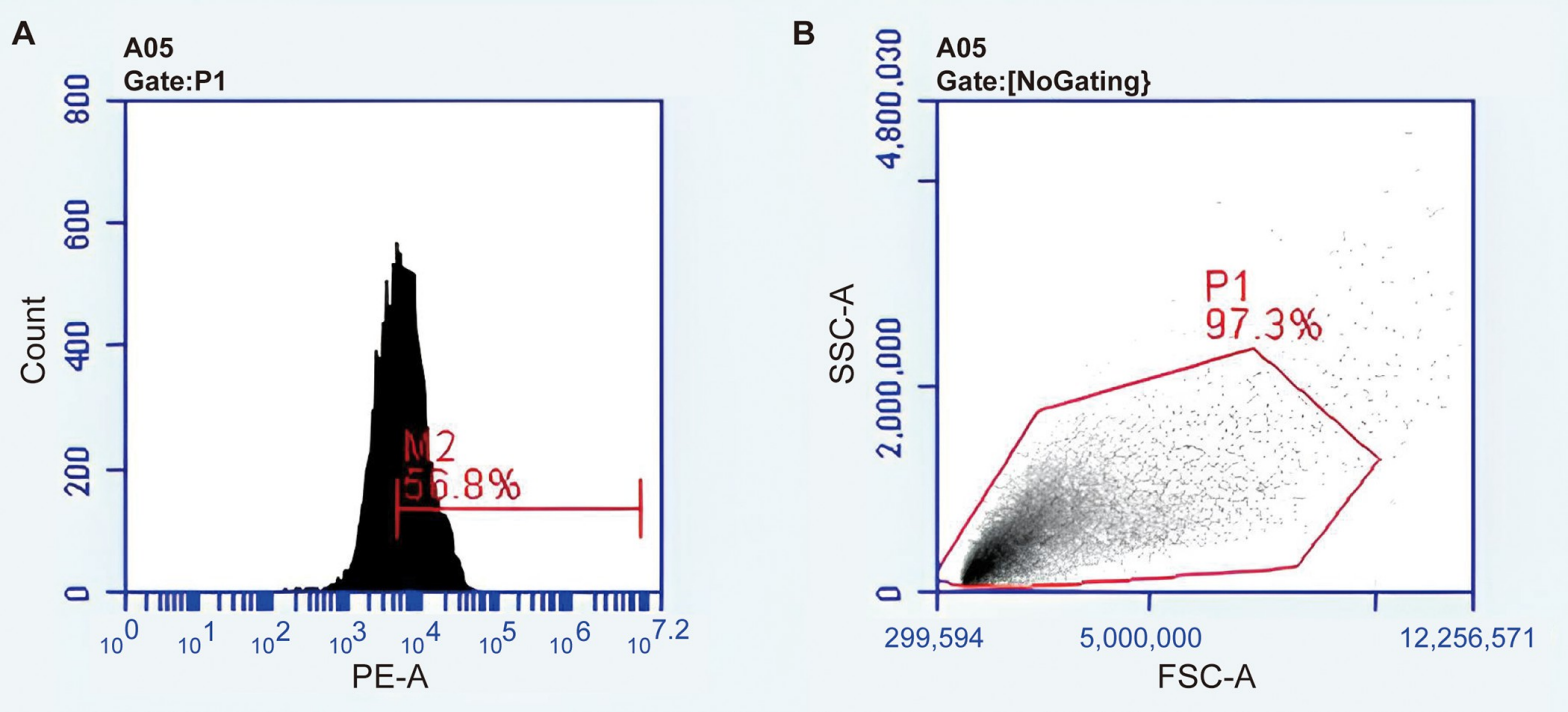
The images of flow cytometry.

We used Lgr5-EGFP-CreERT2/Sox2-CreERT2/Rosa26-tdTomato transgenic mice with postal day (P)0 to P3 to isolate the Lgr5+ HC progenitors. The newborn mice were disinfected by spraying 75% alcohol on the whole body and then put on the ice surface. After entering the state of anesthesia, the mice were decapitated and the cochlea was removed. Then dissect the cochlea in cold 1 × HBSS (Gibco) and transfer it to 200 μl 1 × PBS in 1.5 ml Eppendorf tubes and incubate the tissue in 100μl 0.25% trypsin-EDTA (Gibco) for 8 min at 37°C. Next add 100μl trypsin inhibitor (Gibco, R007100) to stop the digestion and trisect the tissue into a single cell suspension using 100μl complete medium. Finally, cells were filtered through a 40 μl strainer (BD Biosciences, 21008–949) to clear clumps, and the EGFP+ cells were sorted on a BD FACS Aria III (BD Biosciences) (Fig 2).

### Differentiation assay

**Fig 3:**
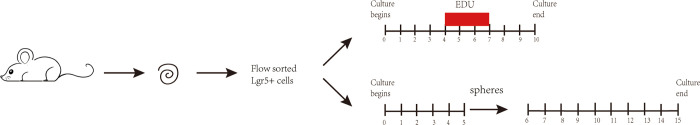
Experiment process. We used the Lgr5-EGFP-CreERT2 transgenic mice to get the basilar membrane of Cochlea and separated Lgr5+ progenitors. Then in direct differentiation assay, we cultured the sorted Lgr5+ cells at 50 cells/μl and added EdU from day 4 to 7. The total culture time was 10 days. In the sphere differentiation assay, we cultured the sorted Lgr5+ cells into spheres and then plated the spheres into silica beads. The total culture time was 15 days.

For the differentiation test, we differentiated both flow-sorted cells and spheres. In the cell differentiation test, flow-sorted Lgr5+ progenitor cells were cultured to a density of 50 cells/μl on laminin-coated (Sigma, L2020) self-assembled silica beads(SB) using DMEM/F12 medium with 1% N2 (Invitrogen, 17502–048), IGF (50 ng/ml, Sigma, I8779),2% B27 (Invitrogen, 17504–044), β-FGF (10 ng/ml, Sigma, F0291), EGF (20 ng/ml; Sigma, E9644), heparan sulfate (20 ng/ml, Sigma, H4777), and 0.1% ampicillin (Sigma, A9518-25G) for 10 days. From day 4 to day 7, EdU (10 μM, Invitrogen, C10340) was added to the cultures (Fig 3).

In the sphere-forming differentiation test,flow-sorted cells were diluted to 5 cells/μl in DMEM/F12 medium with 2% B27 (Invitrogen, 17504–044), IGF (50 ng/ml, Sigma, I8779),1% N2 (Invitrogen, 17502–048), β-FGF (10 ng/ml, Sigma, F0291), EGF (20 ng/ml; Sigma, E9644), heparan sulfate (20 ng/ml, Sigma, H4777), and 0.1% ampicillin (Sigma, A9518-5G) and cultured in Costar ultra-low attachment dishes (Costar, 3599). After 5 days, the spheres were plated on laminin-coated self-assembled silica beads (SB) and cultured in DMEM/F12 medium with EGF (20 ng/ml; Sigma, E9644),1% N2 (Invitrogen, 17502–048), IGF (50 ng/ml, Sigma, I8779), heparan sulfate (20 ng/ml, Sigma, H4777), β-FGF (10 ng/ml, Sigma, F0291), 2% B27 (Invitrogen, 17504–044) and 0.1% ampicillin (Sigma, A9518-5G) for10 days (Fig 3).

### Immunostaining

The cells were fixed in 4% paraformaldehyde for 1 h at room temperature, washed three times with 1 × PBST, and incubated with blocking medium(1% BSA,1% Triton X-100,10% heat-inactivated donkey serum, and 0.02% sodium azide in PBS at pH 7.2) for 1 h at room temperature. Then the primary antibody with PBT1 (1% BSA,10% Triton X-100,5% heat-inactivated goat serum, and 0.02% sodium azide in PBS at pH 7.2) and incubated with samples overnight at 4°C. The samples were washed three times with 1×PBST and incubated for 1 h at room temperature with PBT2 (1% BSA and 0.1% Triton X-100 at pH 7.2) diluted with a secondary antibody. And then washed the samples three times with 1 × PBST and fixed them on slides in DAKO. Cells were imaged with an LSM 700 confocal microscope. The antibodies used in this test were anti-myosin7a (Proteus Bioscience, #25–6790, 1:1000 dilution), Alexa Fluor R 555 donkey anti-rabbit IgG (H+L; Invitrogen, A-31572, 1:400 dilution) and DAPI(1:1000 dilution). Cell proliferation was determined with the Click-it EdU imaging kit (Invitrogen).

## Results

### Direct differentiation assay

**Fig 4:**
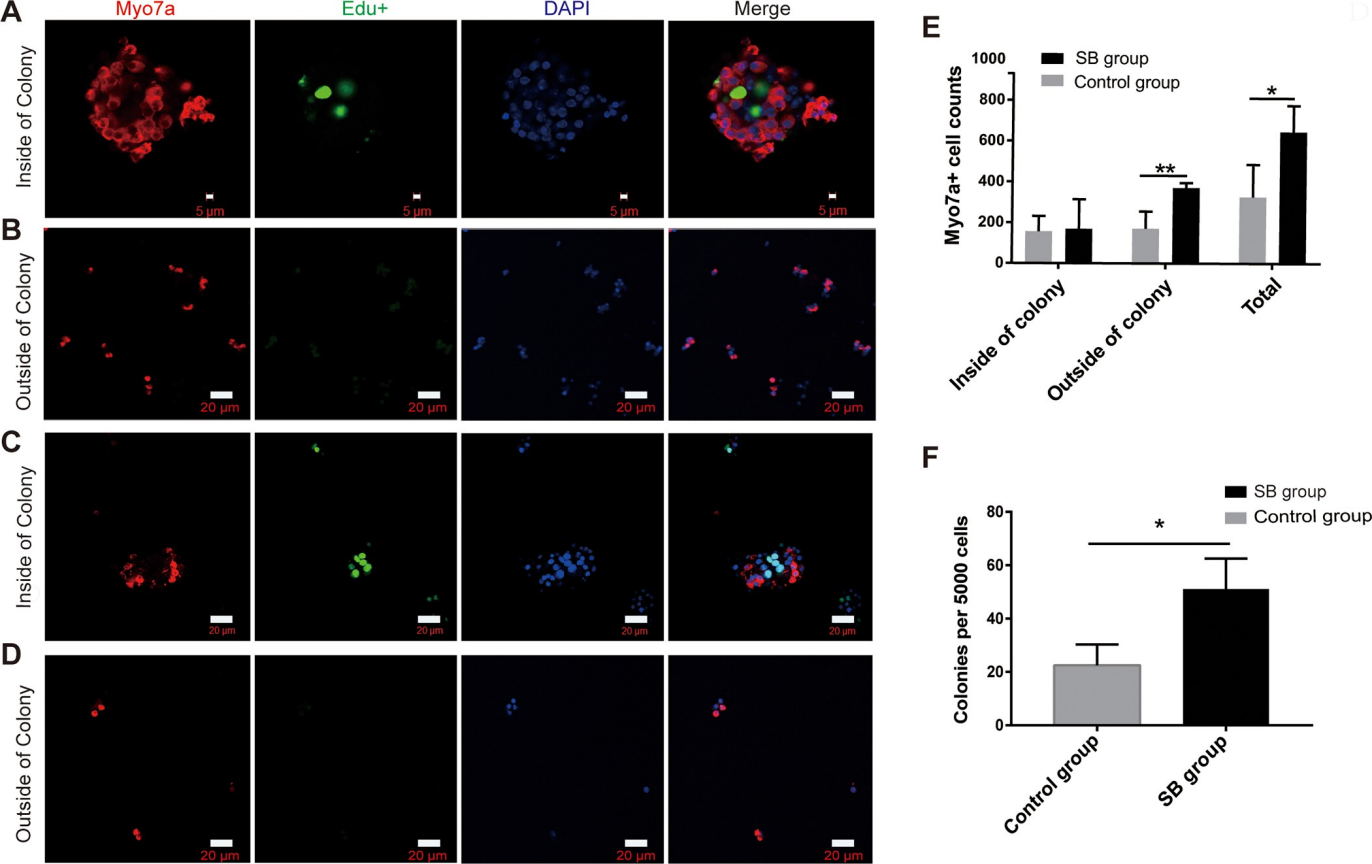
Immunofluorescence figures in the direct differentiation assay. (A)In the SB group, Lgr5+ progenitors generated a large number of Myo7a+ cells on the inside of the colony, and some of them were labeled with EdU. (B)In the SB group, Lgr5+ progenitors generated more Myo7a+ cells on the outside of the colony. (C)In the control group, Lgr5+ progenitors generated few number of Myo7a+ cells on the inside of the colony, and some of the cells were labeled with EdU. (D)In the control group, Lgr5+ progenitors generated few Myo7a+ cells on the outside of the colony. (E)Both total and outside of the colony, in the SB group Lgr5+ progenitors formed more Myo7a+ cells compared with the control group. (F)The number of colonies in each well per 5,000 cells. The Lgr5+ progenitors formed more colonies in the SB group than in the control group.

In the direct differentiation assay, we cultured 5,000 cells in laminin-coated SB at a density of 50 cells/μl for 10 days in a serum-free medium. Slides were used in the control group. We added 10 μM EdU to the culture medium from day 4 to 7 to label the mitotically regenerated HCs. After 10 days of culture, we counted the colonies per 5,000 cells in each well under the microscope. The SB group consisted of 51 colonies, while the control group consisted of 23 colonies. There were statistically significant differences between the SB groups and the control group (*p < 0.05). In the next step in the experiment, the cells were immunostained with the HC marker Myo7a. It was discovered that the SB group produced a greater number of hair cells in comparison to the control group. (*p < 0.05). Especially, in the SB group, Lgr5+ progenitors generated significantly more Myo7a+ HCs outside of the colony than in the control group (**p < 0.01). But there was no difference inside the colony between the two groups(P>0.05). Meanwhile, we found that Myo7a+/EdU+ cells were consistently in the minority both inside and outside the colonies in the two groups (Fig 4).

### Sphere differentiation assay

**Fig 5:**
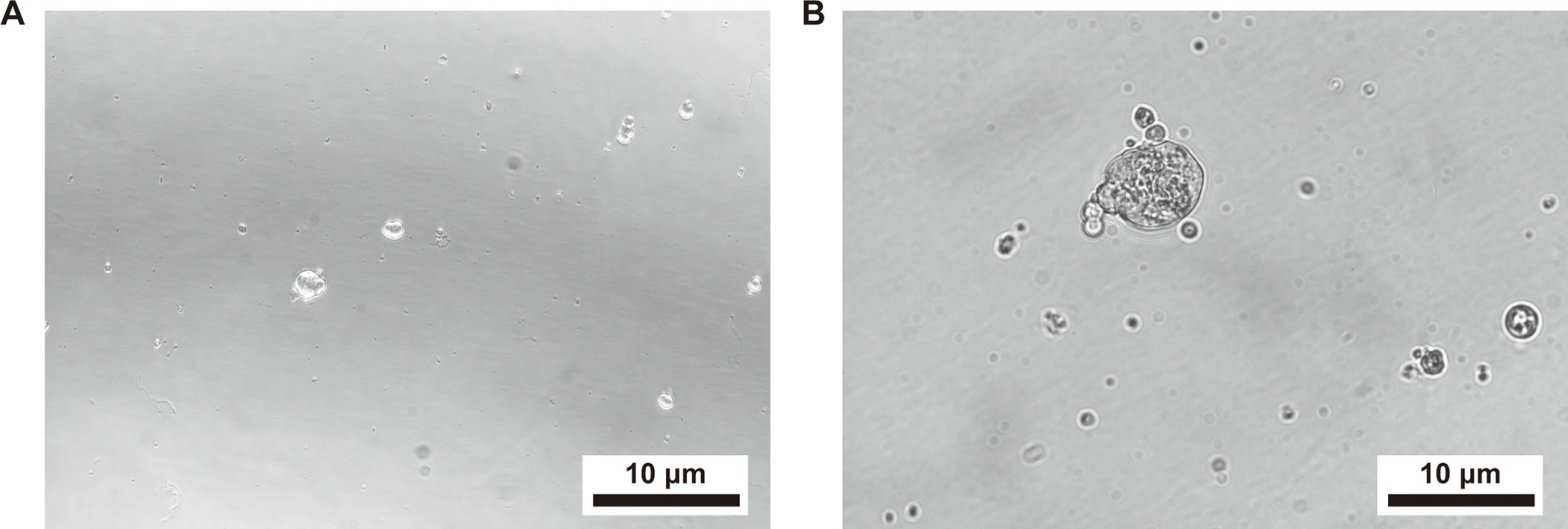
Live cell workstation images. (A) shows the Lgr5+ progenitor cells. (B) shows the sphere formed by Lgr5+ progenitor cells.

**Fig 6:**
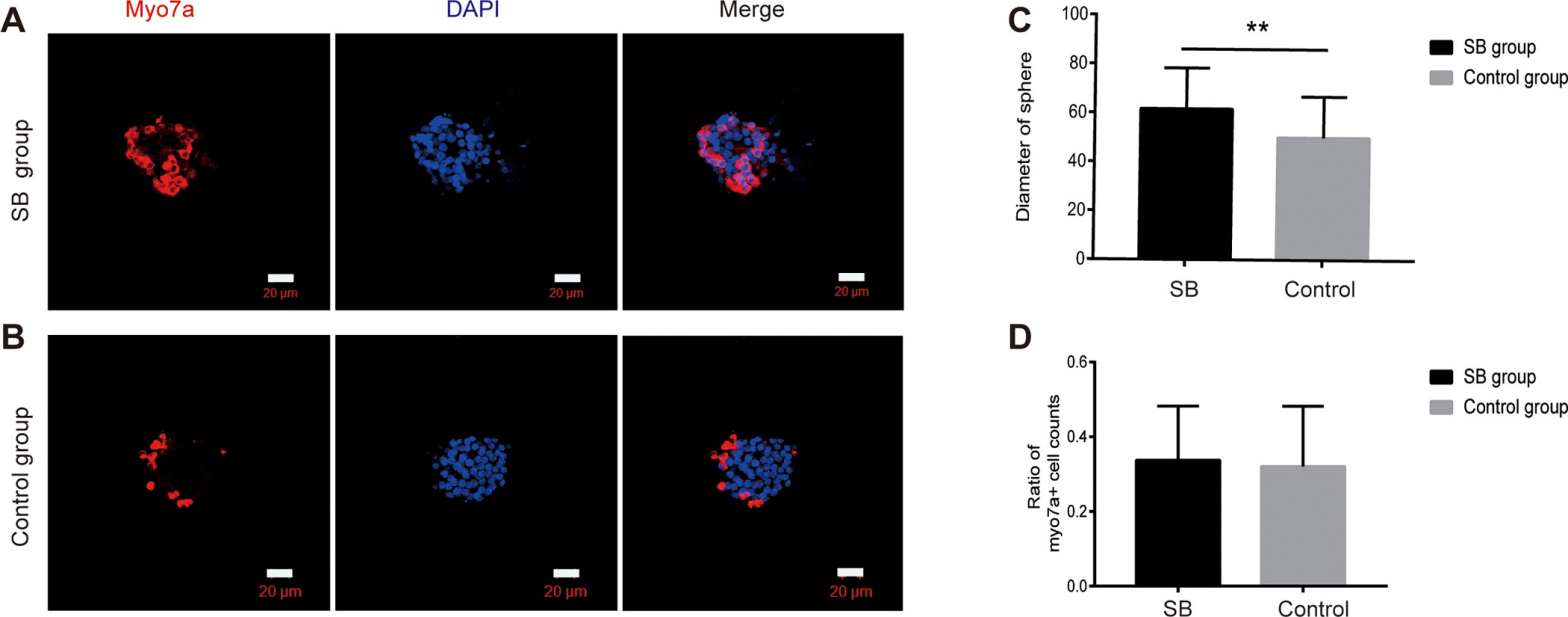
Immunofluorescence figure in the sphere differentiation assay. (A、B)In the sphere differentiation assay, Lgr5+ progenitors formed much the same Myo7a+ cells in the SB group and control group. (C)In the sphere differentiation assay, the diameter of spheres in the SB group was larger than the control group. (D)In the sphere differentiation assay, the ratio of myo7a+ cell counts in the sphere was not obvious between the two groups.

Sphere-forming assays have been used in many studies to assess cell proliferation [21,22]. The objective of the sphere differentiation assay was to determine the regenerative capacity of Lgr5+ progenitors for HCS and spheres on SB. After flow cytometry, we cultured 500 cells at a density of 5 cells/μl in a Costar ultra-low attachment plate for 5 days (Fig 5). We found that the Lgr5+ progenitor cells are capable of forming spheres. Then the spheres were transferred onto laminin-coated SB and cultured for 10 days. In the same way, slides were used in the control group. The method of cell culture was the same as the direct differentiation assay. After 15 days of culture, we measured the diameter of the spheres prior to immunofluorescence and then calculated the ratio of myo7a+ cell counts. We found that the diameter of spheres in the SB group was significantly larger than those of the control group (**p < 0.01). However, the difference in the ratio of myo7a+ cell counts was not apparent (P>0.05) (Fig 6).

## Discussion

In the mature mammalian cochlea, hair cells and supporting cells remain inactive and do not regenerate hair cells spontaneously, so severe hair cell loss leads to permanent hearing loss [23,24].Lgr5 is a stem cell marker found in various bodily, such as the stomach, hair follicles, liver, pancreas, and cochlea [4,5,7,9,25–28].Lgr5+ cells have also been demonstrated to be potential stem cells responsible for the regeneration of the liver, pancreas, and stomach after injury[29].

Studies have shown that Lgr5+ cells can differentiate into hair cells in the cochlear and utricle as progenitor stem cells [30]. In the newborn cochlea, Lgr5+ cells showed the capacity to regenerate spontaneously after injury [2,3].

The nano topological cues are an emerging field of in vivo research because they stimulate the alteration of cell morphology and cell behavior such as adhesion, proliferation, differentiation, apoptosis, and migration [31]. Silica nanobeads can be used as nano-topological tools for cancer cell research [32]. Recently studies have shown that silica beads can promote neurite outgrowth, making it useful in neuroregeneration and neuroprosthetics [19]. However, there have been no studies on the effect of silica beads on inner ear stem cell behavior. In this study, we investigated the differentiation of Lgr5+ progenitor cells on silica beads.

In this study, we sorted the Lgr5+ progenitors from transgenic mice using flow cytometry and cultured them on silica beads. After being cultured for 10 days, new sensory cells were formed and displayed specific markers for hair cells, such as myo7a. We observed that the experimental group regenerated a greater number of hair cells and colonies than the control group through direct differentiation. It was demonstrated that self-assembled silica beads were not toxic to inner ear stem cells in mice and could promote the differentiation of Lgr5+ progenitor cells in vitro. However, the specific mechanism is still unclear and needs to be further studied.

In the direct differentiation assay, we found that in the SB group, Lgr5+ progenitors generated significantly more Myo7a+ HCs outside of the colony compared to the control group. There was no significant difference in the number of Myo7a+ hair cells inside the colony between the two groups. We speculated the possible cause was related to the area of material contact with cells. Outside of the colony, the cells are singly attached to the material. But on the inside of the colony, only a few cells in the cell sphere can make direct contact with the material surface.

In the direct differentiation assay, we found that the SB group regenerated more colonies than the control group. In the sphere differentiation assay, the diameter of spheres in the SB group was larger than that in the control group. The results showed that self-assembled silica beads also had a certain promoting effect on the proliferation of Lgr5+ progenitor cells in vitro.

These silica beads as a topological material have been studied for many years. Previous studies have primarily focused on the effects of materials on neurons, investigations specifically targeting Lgr5+ cells are lacking. Our study represents the first attempt to examine the effect of silica beads on Lgr5+ cell differentiation. However, our investigation did not delve into the underlying mechanism. Related studies have shown that in hippocampal neurons, self-assembled silica beads stimulate intracellular signaling pathways through mechanotransductions of cytoskeletal structure [18]. The mechanism of action on mouse inner ear stem cells remains unclear, which is the focus of our subsequent studies.

In conclusion, our study demonstrate that the self-assembled silica beads can regulate the growth of mouse inner ear stem cells in vitro. It provides important information for the basic research of inner ear stem cells and is expected to become a meaningful research material.

## Supporting information

S1 Data (DOCX)
