# Supplementary material for: The differentiation of Lgr5+ progenitor cells on nanostructures of self-assembled silica beads
Source: PLoS One. 2024 Jul 12;19(7):e0304809. doi: 10.1371/journal.pone.0304809 (PMC11244819; doi:10.1371/journal.pone.0304809)
Supplement: S1 Data — (DOCX) [file pone.0304809.s001.docx]

**Fig4-E: Myo7a+ cell counts**

|  | Control group | SB group | P |
| --- | --- | --- | --- |
| Inside of colony(mean) | 156.00 | 169.00 | 0.914 |
| Outside of colony(mean) | 170.00 | 370.25 | 0.008 |
| Total(mean) | 326.00 | 645.50 | 0.043 |

T-test

|  | Control group | SB group |
| --- | --- | --- |
| Mean value | 22.50 | 51.00 |

**Fig4-F: Colonies per 5000 cells**

1. test P=0.033

**Fig6-C: Diameter of sphere**

|  | SB group | Control group |
| --- | --- | --- |
| Mean value | 61.60 | 50.04 |

T-test P=0.009

**Fig6-D: Ratio of myo7a+ cell counts**

|  | SB group | Control group |
| --- | --- | --- |
| Mean value | 0.352 | 0.346 |

T-test P=0.900
